# K-space polarimetry of bullseye plasmon antennas

**DOI:** 10.1038/srep09966

**Published:** 2015-04-30

**Authors:** Clara I. Osorio, Abbas Mohtashami, A. Femius Koenderink

**Affiliations:** 1 Center for Nanophotonics, FOM Institute AMOLF, Science Park 104, 1098 XG Amsterdam, The Netherlands

## Abstract

Surface plasmon resonators can drastically redistribute incident light over different output wave vectors and polarizations. This can lead for instance to sub-diffraction sized nanoapertures in metal films that beam and to nanoparticle antennas that enable efficient conversion of photons between spatial modes, or helicity channels. We present a polarimetric Fourier microscope as a new experimental tool to completely characterize the angle-dependent polarization-resolved scattering of single nanostructures. Polarimetry allows determining the full Stokes parameters from just six Fourier images. The degree of polarization and the polarization ellipse are measured for each scattering direction collected by a high NA objective. We showcase the method on plasmonic bullseye antennas in a metal film, which are known to beam light efficiently. We find rich results for the polarization state of the beamed light, including complete conversion of input polarization from linear to circular and from one helicity to another. In addition to uncovering new physics for plasmonic groove antennas, the described technique projects to have a large impact in nanophotonics, in particular towards the investigation of a broad range of phenomena ranging from photon spin Hall effects, polarization to orbital angular momentum transfer and design of plasmon antennas.

An ultimate goal of nanophotonics is to engineer single nanostructures, or clusters of them, capable of precisely manipulating the propagation, emission and absorption of light. A large interest in this capability stems on one hand from projected applications of plasmonics^1^ and metamaterials^2^,^3^ in domains ranging from improved photovoltaics^4^, efficient solid-state lighting^5^ and on-chip optical components^1^ to the improvement of research tools in spectroscopy and microscopy at the single molecule level^6^. On the other hand, the fields of plasmonics, metamaterials, and metasurfaces^1^,^2^,^3^ continue to surprise both with new insights in the peculiar solutions of Maxwell's equations when exotic material responses are introduced, and in the parallels with solid-state phenomena such as the spin hall effect^7^,^8^,^9^,^10^,^11^,^12^ or topological insulator physics^13^.

The behavior of any single nanostructure in response to an incident optical wave is most generally described by a so-called *t*-matrix^14^,^15^,^16^, also known as scattering matrix or generalized transmission function. In the case of scattering, the *t*-matrix completely specifies the far-field distribution for any incident field, including polarization, amplitude, phase, and k-distribution of both fields^14^,^15^,^16^. Experimental techniques for the characterization of nanostructures, such as bright and dark field microscopy or NSOM, measure different subsets of the transmission function. However, there is no single technique that can map the complete transformation of incident light into the far field.

This paper introduces high-NA k-space polarimetry^17^,^18^,^19^,^20^ as a technique to measure the response of single scatterers to incident fields with different polarizations. This technique combines a Fourier microscope^21^,^22^, capable of mapping the k-vector distribution of scattered radiation, with a polarimeter^23^,^24^,^25^ that measures the full polarization state for each wave vector. For a given incident k-vector distribution, a k-space polarimeter measures all information encoded in the transmission function of a scatterer across an entire microscope back aperture, up to an over-all phase.

In order to demonstrate k-space polarimetry we consider bullseye antenna scatterers (BEs), consisting of periodic grooves concentric to a circular hole in a plasmonic metal film, Fig. 1 (a). These antennas are among the simplest, most widely used, and best understood plasmonic structures that scatter light directionally^26^,^27^,^28^,^29^. Furthermore, bullseye antennas are widely studied for their ability to impart directionality to the fluorescence of fluorophores residing in the central aperture^30^. While the role of wavelength and antenna design on field enhancement and directionality is well understood^31^, the polarization state of the light scattered by BEs has been only partially characterized^32^,^33^,^34^,^35^. Here we use k-space polarimetry to measure the angle-resolved polarization state of the scattering of bullseyes under different illuminations. Our results show strong linear-to-circular polarization conversion at off-normal scattered wave vectors, showing that even a structure as ubiquitous as a bullseye antenna still contains surprising physics, relating to the emerging field of controlling spin-orbit coupling for photons^7^,^8^,^9^,^10^,^11^,^12^.

## K-space polarimetry

At the basis of our k-space polarimeter is a conventional microscope, with a “Bertrand” or “Fourier” lens. A Fourier microscope exploits the fact that the back focal plane of a microscope objective provides access to the entire distribution of k-vectors collected by it, which can be directly mapped onto a CCD camera chip. Fourier imaging has been applied, for instance, to image the radiation pattern of single emitters to determine their dipole moment^21^,^22^ and to map the directivity optical antennas impart to emitters^30^,^36^,^37^,^38^. Fourier microscopes have been also used in scattering experiments on single nanostructures^39^ and nanostructure arrays^40^,^41^,^42^,^43^. The back focal plane in a Fourier microscope retains full information regarding momentum, but also in other degrees of freedom such as energy (frequency) and polarization. Accessing this information requires additional analyzers. For instance, energy resolved radiation patterns have been measured using spectrometers^44^ or gratings^45^ to disperse Fourier images.

Regarding polarization, Fourier microscopy measurements have been reported with a single linear polarizer as analyzer^39^,^46^, which only partially interrogates the polarization state of the scattered field. Given the nature of polarization, it is fundamentally impossible to retrieve the full state from linear-polarization measurements. Moreover, the very strong refraction of rays in high NA aplanatic lenses means that imaging through linear analyzers behind the microscope objective does not correspond to a natural polarization basis. Indeed, in the beam behind the objective, the basis of *s* and *p*-polarization applicable to the spherical wave emanating into the far field from a scatterer in the object plane converts to radial and azimuthal polarization, and not into orthogonal Cartesian polarizations. Thereby a simple linear polarization analysis is incomplete, and impractical.

Polarimeters perform complete polarization measurement, that is measurements that allow retrieving the Stokes parameters *S*_0_, *S_1_, S*_2_ and *S*_3_ In this work we place a polarimeter in a Fourier microscope, to determine the polarization state for each scattered k-vector. We use a rotating-plate polarimeter composed by a quarter wave plate (QWP) followed by a linear polarizer (LP)^23^,^24^,^25^. These two elements act as a linear polarizer when their optical axes are aligned and as a circular polarizer when the angle between their optical axes is *π*/4. If *I_α,β_* is the intensity measured after rotating the QWP by an angle *α* and the LP by an angle *β* (subscript labels expressed in degrees) with respect to the *x* axis, the Stokes parameters are given by
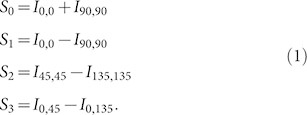
Thus a total of six measurements are used to retrieve the four Stokes parameters. The first Stokes parameter, *S*_0_, corresponds to total intensity. The other three parameters are given by the difference between intensities transmitted by orthogonally orientated polarizers: horizontal and vertical for *S*_1_, diagonal plus and minus for *S*_2_, and right and left-handed circular for *S*_3_.

Since the Stokes parameters fully describe polarization, any other figure of merit for polarization can be retrieved from them. For instance, the total degree of polarization *DP* and the degrees of linear *DLP* and circular polarization *DCP* are given by
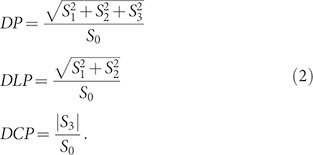


This capability of a polarimeter to determine how much of a beam is actually polarized would be especially useful in plasmon-enhanced fluorescence experiments, where coupling to a plasmon resonance may impart polarization even to randomly oriented dipole emitters. Other intuitive, and commonly used, figures of merit for polarization that are easily obtained from the Stokes parameters include the amount of *s*- and *p*-polarized light, or the ellipticity and orientation of the polarization ellipse.

## Experimental setup

Figure 2 shows our setup with its two main components: a home-built Fourier microscope (b) and a rotating-plate polarimeter (c). As a light source, we use a supercontinuum laser (Fianium) filtered by an acousto-optical tunable filter (AOTF) and a 20 nm band pass filter centered at 750 nm. A linear polarizer and a quarter wave plate set the input beam polarization, Fig. 2(a). A 10 × objective weakly focuses the light on the sample and a 60 × objective (NA = 0.7) collects the resulting radiation. The presented technique is not limited to these choices: both illumination and detection sides can encompass any available objective NA. Moreover, a full polarimetric k-vector mapping on the incident side is possible by scanning a mildly focused input beam in the back focal plane of the input objective^47^ On the collection side, light passes through a spatial filter in an intermediate image plane to isolate light scattered only by a single nanostructure. The spatial filter is composed of a 1:1 telescope (2*f*_telescope_ = 100 mm) and a 300 *μ*m pinhole, equivalent to about 20 *μ*m on the sample. To obtain Fourier images, i.e., to image the back focal plane of the objective onto a CCD camera (Photometrics CoolSnap EZ), we use a *f*_Fourier_ = 200 mm lens placed at a distance 4*f*_telescope_ + *f*_Fourier_ from the back focal plane, followed by a *f*_tube_ = 200 mm tube lens. The rotating-plate polarimeter consists of a broadband quarter wave plate (Thorlabs AQWP-600) and a linear polarizer (Thorlabs LPVIS100), and it is placed just before the tube lens.

**Figure 1:**
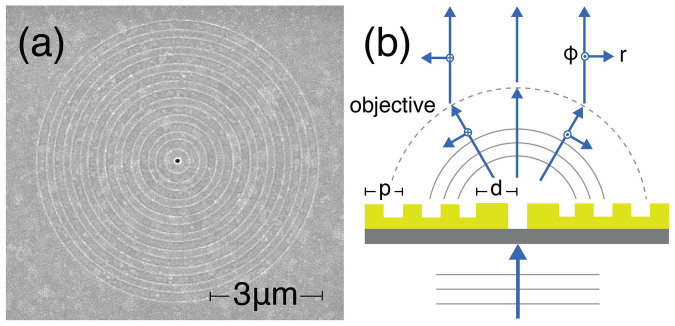
We study the polarization properties of the scattering of bullseye antennas. (a) Shows a scanning electron micrograph of 440 nm pitch antenna. (b) The spherical wave emanating into the far field from a scatterer in the object plane, naturally described in the *S* and *P*-polarization basis, is transformed by the objective into plane waves.

Our sample was fabricated in a 200 nm thick gold film evaporated on a glass cover slip that was first coated with a 5 nm chromium adhesion layer. The bullseye antennas were imprinted on the film with a focused ion beam (FIB) by milling isolated circular holes with a diameter of 210 nm through the metal and engraving 8 grooves of about 60 nm depth that are concentric with the holes. We studied structures with different distances between consecutive grooves (pitch *p*) and between the central hole and the first groove (*d*). We present results for two structures BE_440_ with *p* = 440 nm and *d* = 330 nm, shown in Fig. 1(a) and BE_600_ with *p* = 600 nm and *d* = 600 nm. In all cases the width of each groove was half the pitch. In our experiments the structure is immersed in water, so as to provide a scatterometry dataset that directly compares to the conditions also used in experiments on fluorescence enhancements in Ref. 30.

## Measurements and results

**Figure 2:**
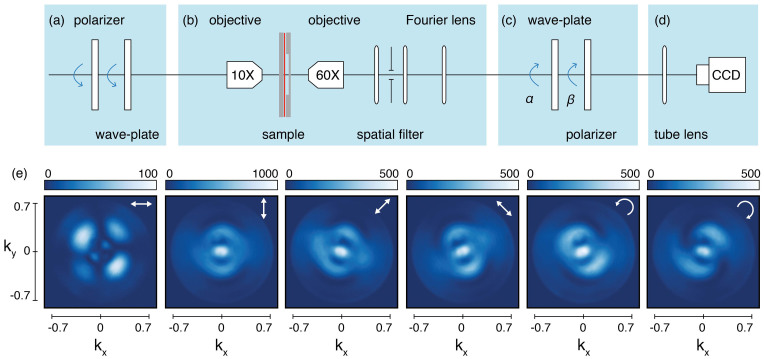
(a)–(d) Experimental setup. The polarization of the incident light is prepared in (a). Combining a Fourier microscope (b) and a rotating-plate polarimeter (c) it is possible to measure the angle-resolved Stokes parameters of the scattered light in the CCD camera in (d). (e) Consecutive measurements with six different settings of the polarimeter are required to retrieve the Stokes parameters. The figure shows these measurements for the bullseye antenna BE_440_ illuminated with vertically polarized light. The arrows indicate the polarization transmitted by the polarimeter at each measurement and the circle indicates the NA. The measurements are performed with constant incident power and the color bar indicates the number of counts in the camera integrating over 0.1 s. Notice that the first two panels have a very different scale as a result of the polarization of the measured scattering.

In order to retrieve the Stokes parameters of the light scattered by each structure, we measured its k-space distribution with six settings of the polarimeter given by Eq. 1. Figure 2(e) shows the set of measurements for BE_440_ illuminated with vertically polarized light. The k-space distribution is a circular disk on the CCD chip because of the Abbe sine condition for microscope objective design. The center of this disk corresponds to the microscope optical axis, i.e., |**k**_||_| = 2*π*/*λ* sin*θ* = 0. The outer rim corresponds to the objective NA and the distance *d* from image center to edge relates to angle as *d* ∝ sin*θ* (angle in air)^39^,^48^,^49^. As mentioned before, the back focal plane images directly correspond to the **k**_||_ distribution of scattered light.

The raw data in Fig. 2(e) reveal the characteristic pattern for the light scattered by bullseye antennas first reported by Lezec et al.^27^ The intensity is strongly peaked in a narrow lobe around the forward direction, which is surrounded by a fringe at sin*θ* ≈ 0.2. Similar behavior has been previously shown using conventional rotation-stage set-ups^35^ and is usually explained by diffraction. At the measurement wavelength, the groove period matches a 2nd order Bragg condition for the surface plasmon supported at the grooved Au interface. Coherent addition of plasmons scattered out as light into the far field by the grooves thereby leads to a directional beam. The highest intensity is observed when analyzing the scattered light in the polarization-conserving channel (*I*_90,90_), with an almost 10:1 ratio to the cross polarized channel (*I*_0,0_). However, even if most of the scattering retains the polarization of the incident light, the intensity of horizontally polarized light *I*_0,0_ is non negligible. In part this is expected, since plasmons launched at the hole are subsequently radiated by the grooves. Hence the *p*-polarized nature of surface plasmons must appear in the scattered light. Indeed, the presence of the grooves strongly raises the cross polarized intensity to a level at least 3 orders of magnitude higher than obtained for a single hole (measurement not shown). The raw data also reveal more subtle and surprising details. For instance, the raw measurements using circular polarization analysis show that scattered light is handed for particular wave vectors, even though the sample and the illumination are mirror symmetric. In particular, at off-normal angles lobes with left- and right-handed polarization appear at mirrored scattered wave vectors. The slight asymmetry in shape and intensity of different lobes in the same measurement is due to small misalignments of the angular position of the polarimeter elements, which we can determine only with a precision of ± 2°. Other sources of error in this kind of measurements include mirrors and birefringent optical elements such as some microscope objectives.

**Figure 3:**
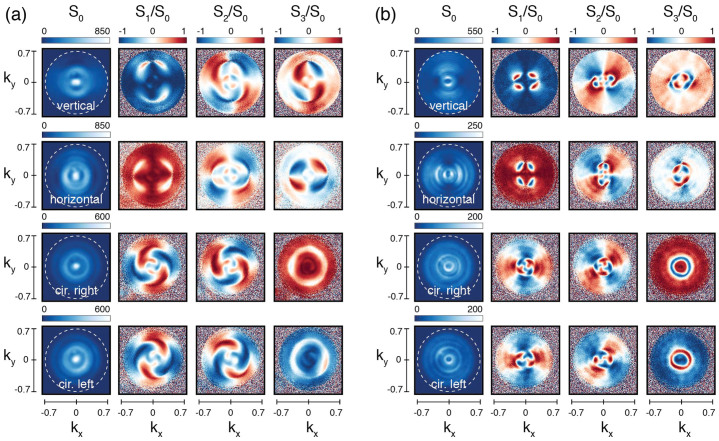
Angle-resolved Stokes parameters for BE_440_ in (a) and BE_660_ in (b), under linearly (first two rows) and circularly polarized light (second two rows). The Stokes parameters *S*_1_, *S*_2_, and *S*_3_ are normalized to the total scattered intensity *S*_0_. The color bar on *S*_0_ corresponds to counts in the CCD camera in 0.1 s. Differences in the scales of *S*_0_ correspond to differences on the intensity at which each set of six measurements were performed.

We now discuss the Stokes parameters retrieved from the raw data, which provide complete (and non-redundant) information on the angular-dependent features of the polarization state of the scattered light. Figures 3 (a) and (b) show the Stokes parameters of the light scattered by the two antennas, BE_440_ and BE_600_ respectively, for four distinct input polarizations. In these figures, each row corresponds to a different incident polarization (vertical, horizontal, right and left handed circular), while each column represents different Stokes parameters. The first column shows the total intensity, *S*_0_, while the last three columns show the parameters *S*_1_, *S*_2_ and *S*_3_ normalized to the total intensity *S*_0_. The angle-dependent intensity summed over all polarization contributions, *S*_0_, is directly comparable to literature reports on the physics of bullseye antennas^30^,^35^. Since we consider a cylindrically symmetric structure excited along the symmetry axis, solely the incident polarization should determine the symmetry of scattering patterns in *S*_0_ as confirmed by Fig. 3 (a) and (b). While circularly polarized incident light results in rotationally invariant radiation patterns, this invariance is lost when illuminating with linearly polarized light since it breaks the symmetry of the system. The resulting elongated pattern rotates with the incident linear polarization, as was shown earlier in ref. 35 when studying the polarization of a single line (*k_y_* = 0) in a Fourier image.

Regarding the polarization properties of the scattered light, our measurements show that at |*k*_||_| = 0 the scattering always retains the polarization of the incident field as required by the cylindrical symmetry of the system (structure plus incident and scattered wave vectors). At scattering vectors |*k*_||_| ≠ 0 the incident polarization is not trivially translated into the polarization of the scattering and, for instance, there are well defined regions where input polarization and scattering are *orthogonally* polarized. In the case of linear incident polarization, these regions appear as four elongated lobes in *S*_1_/*S*_0_ in the first two rows of Fig. 3 (a) and (b). For incident circular polarization, annular regions appear where the scattering is oppositely handed to the incident field, as shown by *S*_3_/*S*_0_ in the last two rows of each figure. In both cases, at scattering angles surrounding these regions, the structures convert linear polarization into circular/elliptical polarization and vice versa. Thus, even these simple, cylindrically symmetric structures show strong polarization conversion at specific wave vectors, a phenomenon of recent interest in the field of controlling spin-orbit coupling of light by nanophotonic structures^7^,^8^,^9^,^10^,^11^,^12^.

**Figure 4:**
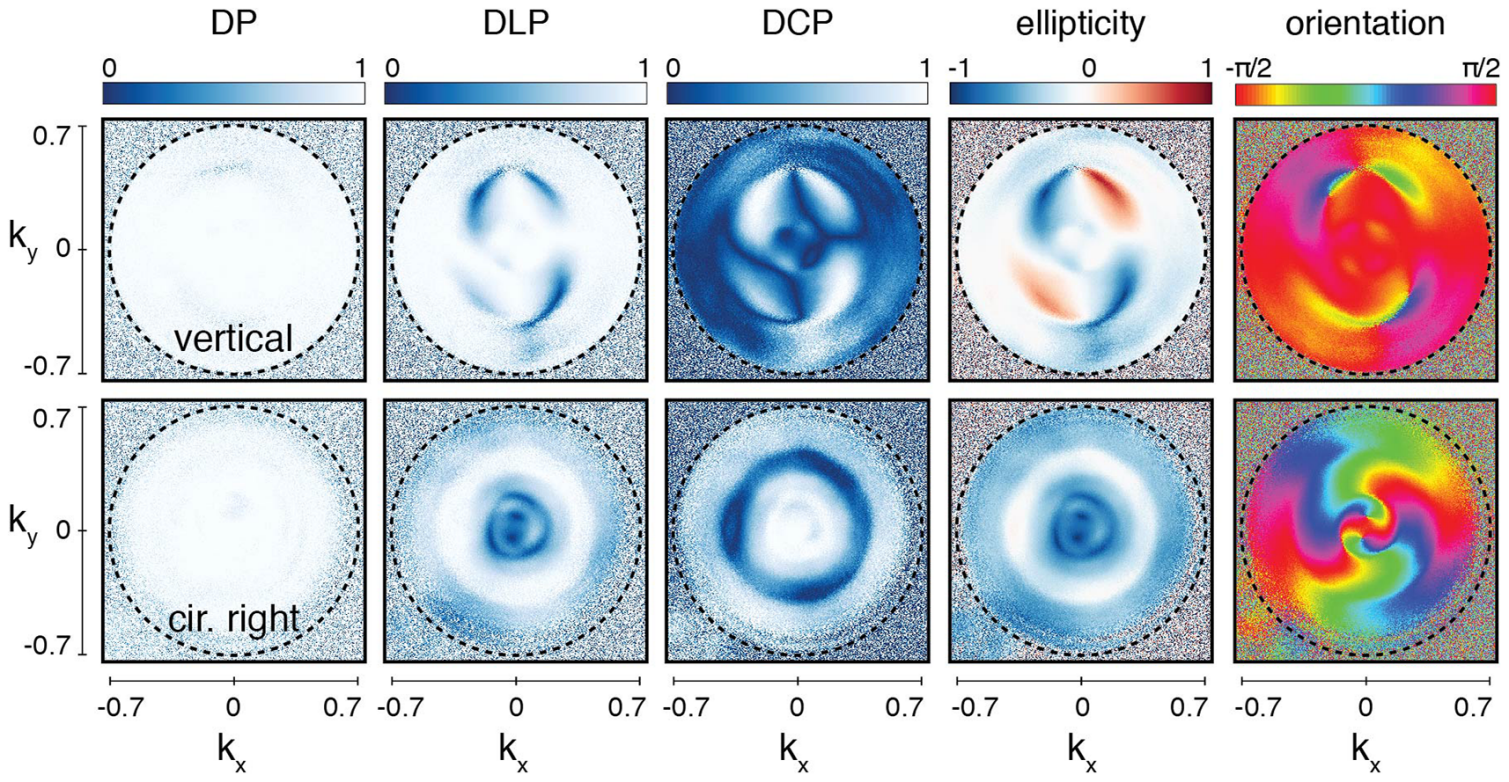
Different figures of merit for polarization for BE_440_ illuminated with vertically polarized light (first row) and right handed circularly polarized light (second row), retrieved from the Stokes parameter shown in Fig. 3. The first three columns show the degree of polarization *DP*, degree of linear polarization *DLP* and degree of circular polarization *DCP*. The last two columns show the ellipticity *ε* and orientation of the polarization ellipse *ψ* for the fully polarized scattering of the structure.

There are many other bases into which the retrieved polarization information may be cast, for instance to best bring out the geometry of a particular scattering problem or to best suit a researchers intuition. As example, we demonstrate the retrieval of different figures of merit for BE_440_ illuminated with vertically and right-handed circularly polarized light. Figure 4 shows the retrieved degree of polarization *DP*, degree of linear polarization *DLP* and degree of circular polarization *DCP*. As would be expected for a completely coherent scattering process, even though the scattered field presents a complex polarization, the structure does not decrease the degree of polarization of the incident field. The scattering conserves the incident total degree of polarization *DP* = 1 for every k-vector independently of the incident polarization. As shown before, most of the scattered light has a degree of linear (circular) polarization that closely matches the incident light. Conversion from linear to circular polarization and vice versa, occurs in well defined regions where there is a quarter wave phase difference between the light emanating directly from the hole (polarized as the incident field over the entire back aperture) and that radiated by the grooves (radially polarized over the back aperture owing to the *p*-polarized nature of plasmons).

Figure 4 also shows the parameters of the ellipse described by the electric field vector as a function of time, which are a frequently used representation of the polarization state of fully polarized light^25^. The ellipticity *ε*, defined as the ratio of semi-major and semi-minor axis, takes values between 0 for linearly polarized light to ± 1 for right and left handed circularly polarized light, while *ψ* denotes the orientation of the ellipse. The angle *ψ* runs from −*π*/2 to *π*/2, where 0 means that the major axis points along *x*. This representation not only highlights the strong polarization conversion, already evident in the degree of linear and circular polarization, but further allows a detailed tracking of the polarization ellipse orientation. In particular, it unveils the presence of so-called C-points which are polarization singularities where *ψ* is undefined, corresponding to nodes of purely circularly polarized scattering^50^,^51^,^52^,^53^,^54^,^55^.

## Comparison to a theoretical model

The polarization information obtained with k-space polarimetry can serve as benchmark to test models currently used to describe the behavior of bullseye antennas. In particular, here we extend a common simplified, scalar model developed in Ref. 29,35,56 to describe the intensity distribution of scattering by BEs, to also predict all angular features in its polarization content. In brief, the accepted model for scattering by structures in gold consisting of a nanoaperture surrounded by corrugations is that the radiation pattern is composed of two contributions. First, the nanoaperture itself is assumed to radiate into the far field as a point source. Second, plasmons launched at the hole propagate into the film as a circular wave, approximated as 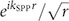, where *k*_SPP_ is the complex plasmon wave vector that accounts for phase accumulation and loss. The surface plasmons subsequently excite the grooves that act as secondary sources radiating out into the far field. In this model, the radiated scalar field observed at an observation point a distance *R* away from the scatterer, and at a viewing angle set by the parallel wave vector |**k**_||_| is proportional to
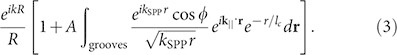


With the complex ratio *A* between the central hole contribution and that of the grooves, and the effective coherence length *l_c_* as free parameters, this scalar model has proven remarkably successful for explaining beaming (i.e., *S*_0_) despite the abstraction of each groove as an infinitely thin radiating circle^29^,^56^,^35^ and the neglect of multiple scattering effects.

To include polarization in this model, we make the following two key modifications. First, we consider the central hole as an in-plane electric dipole that both radiates into the far-field and launches surface plasmon polaritons in the metal air interface (SPP) according to a cos *φ* in-plane angular amplitude distribution (*φ* being the angle between the in-plane dipole and the in-plane wave vector). Second, following^57^, the grooves are modeled as lines of in plane magnetic dipoles tangential to the grooves. We take the in-plane dipole moment induced in the central hole to be directly inherited from the input polarization of the driving field. Finally as radiation pattern for each elementary radiator in the system we use the full dipolar radiation pattern for dipoles above a substrate as derived by Lukosz et al.^58^

As parametric input to the model we use the surface plasmon polariton wave vector calculated from tabulated optical data^59^ and include the groove periodicity as taken from the SEM characterization. The model then depends on several ‘free parameters’. These are the complex ratio (*A*, written as |*A*|e*^iφ^*) of the scattering amplitudes of the central hole and the grooves, the effective coherence length *l_c_* and the effective location of the magnetic dipoles relative to the center of the groove they represent. We parametrize these values through *a*, the effective radius at which the first groove occurs. Since we consider grooves with a 50% duty cycle, the relation between *a* and the actual distance between hole and first groove in the sample is by no means trivial. However, we naturally require that for a given structure scattering patterns for any incidence condition are explained by the same parameter set.

**Figure 5:**
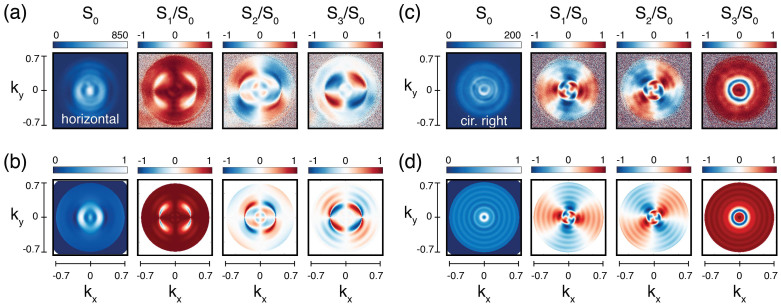
Comparison between measured (a)–(c) and calculated (b)–(d) angle-resolved Stokes parameters in two configurations: (a) and (b) correspond to BE_440_ illuminated with horizontally polarized light, while (b) and (c) correspond to BE_660_ illuminated with right handed circularly polarized light.

Figure 5 shows the comparison between the measured Stokes parameters (a)–(c), and those calculated using the model, (b)–(d). Here we focus on two systems, BE_440_ illuminated with horizontally polarized light (a)–(b) and BE_660_ illuminated with right handed circularly polarized light (c)–(d). As free parameters, for both structures the ratio between the intensity scattered by the central hole and the grooves is taken as |*A*| = 0.1 *μm*^−1^ and *φ* = *π*/2 and the effective coherence length as function of the pitch *p* is *l_c_* = 10*p*. The differences in actual geometry result in *a* = 149 nm for BE_440_ in (b) and *a* = 266 nm for BE_660_ in (d). Figure S1 in the supplementary information shows the calculated patterns for all measurements in Fig. 3. Inspection of the calculation shows that the simple model reproduces all salient features in the data for all input polarizations, and for all output polarizations. These notably include the beaming in *S*_0_, the occurrence of pockets of output polarization orthogonal to the input, as well as the angular regions in which linearly polarized light is converted to circular polarization and vice versa.

It is important to notice that, while the scalar model^29^,^56^,^35^ and the vectorial form of it are very robust for predicting total intensity *S*_0_, the angle dependent polarization features encoded in *S*_1,_ *S*_2_ and *S*_3_ are much more sensitive than the total intensity to the choice of the free parameters. For instance, there is a large range of dipole amplitude choices where the intensity pattern hardly changes, whereas the polarization features vary dramatically, as shown in the supplementary information (Fig. S2). Also, we found that while a good match to the data is obtained for a particular combination of *a*, |*A*| and *φ*, this combination is not unique. This observation allows us to draw two conclusions. On one hand, the fact that the simple, commonly used model is so successful for predicting intensity patterns should in retrospect not be read as a validation *per se* of its input parameters, or of the involved approximations of abstracting the hole to a point and the grooves to infinitely thin circles of secondary radiators. Indeed, we find that good matching of the model to just overall intensity, i.e., *S*_0_ is possible for a very wide range of input parameters. In contrast, matching the full set of Stokes parameters is much more demanding. Thereby we draw as second conclusion that Fourier polarimetry provides experimental signatures that are excellently suited to discriminate between different, improved models for the response of plasmonic bullseyes, and by extension also for spirals, plasmonic crystals, and array antennas.

## Conclusions and perspectives

We have reported a new measurement technique to resolve the polarization state of light scattered by a single nanostructure as function of wave vector across an entire microscope back aperture. The technique combines back focal plane imaging, also known as Fourier microscopy, with a polarimeter consisting of a linear polarizer and quarter wave plate. From just six camera images, all polarization content can be retrieved as we demonstrate for a simple bullseye antenna. Our results evidence some remarkable features of the scattering of BEs. The scattering pattern of BEs strongly depends on the incident polarization. Circular incident polarization result in rotational symmetric patterns while linear polarization results in patterns elongated in the direction of the polarization, as consequence of the *p*-polarized nature of the involved plasmon excitation. While scattered light dominantly retains the polarization of the incident field there are well defined regions at |*k*| ≠ 0 where the polarizations of incident and scattered field are different, even orthogonal to the incident field, or completely converted in helicity from −1 to 0 or + 1.

The reported measurement technique is equally applicable to fluorescent nanostructures in which the total degree of polarization is not unity, and is in fact an important parameter. For instance, while randomly oriented emitters should result in *DP* = 0, once they are strongly coupled to the resonance of a plasmonic nanorod, their emission is expected to inherit the orientation of the rod as dominant polarization. Thus mapping the degree of polarization could be an important quantifier to measure the efficiency with which a nanostructure controls emission polarization. We argue that Fourier polarimetry is an easily implemented, yet extremely sensitive tool to test our understanding of a plethora of dielectric and metallic nanophotonic structures. Beside plasmonics, this technique could be also a useful tool to study molecular orientation in biological samples^60^ or in spin populations^61^, optically induced magnetic order^62^, or as a complement of other imaging techniques such as orientation imaging microscopy^63^.

## Author Contributions

C.I.O. conceived the experiment and performed the measurements. A.M. designed and fabricated the samples. C.I.O. and A.F.K. developed the theoretical model. A.F.K. supervised the project. All authors contributed to the manuscript.

## Supplementary Material

Supplementary Information Supplementary information
