# Supplementary material for: K-space polarimetry of bullseye plasmon antennas
Source: Sci Rep. 2015 Apr 30;5:9966. doi: 10.1038/srep09966 (PMC4415650; doi:10.1038/srep09966)
Supplement: Supplementary Information [file srep09966-s1.pdf]

Supplementary information - K-space polarimetry of bullseye plasmon antennas

Clara I. Osorio, Abbas Mohtashami, and A. Femius Koenderink*

Center for Nanophotonics, FOM Institute AMOLF,

Science Park 104, 1098 XG Amsterdam, The Netherlands

In addition to the cases shown in Fig. 5 of the main text, we calculated the Stokes parameters corresponding to all measurements in Fig. 3. Figure 1 shows the angle-resolved Stokes parameters for BE_{440} in (a) and BE_{600} in (b) illuminated with linearly and circularly polarized light. These calculations were performed fixing the free parameters to $|A| = 0.1\ \mu\mathrm{m}^{-1}$, $\varphi = \pi/2$, $l_c = 10p$, $a = 149$ nm for BE_{440} in (b) and $a = 266$ nm for BE_{600}. These values were retrieved by comparing the calculated to the measured Stokes parameters.

As discussed in the main text, the angular dependent polarization features are more sensitive to the choice of parameters than the total intensity. To illustrate this, Fig. 2 shows the Stokes parameters calculated for BE_{600} under horizontal polarization for (a) different values of a (shown in units of the pitch) and (b) $|A|$. In (a), the total intensity, S_0 shows very similar patterns independently of a, in all cases the expected two-lobe pattern appear at angles close to those predicted from diffraction. In contrast, the angular-dependent features of the polarization strongly vary with a as a result of the different phase acquired by the plasmons while propagating from their origin to the first groove. In the case of the variations as function of $|A|$, Fig. 2 (b) shows the extreme cases of having a scattering completely dominated by the grooves (last row) or by the central hole (first row). As in the previous case, the main features of the intensity distribution S_0 does not change dramatically for relatively close values of $|A|$.

* f.koenderink@amolf.nl

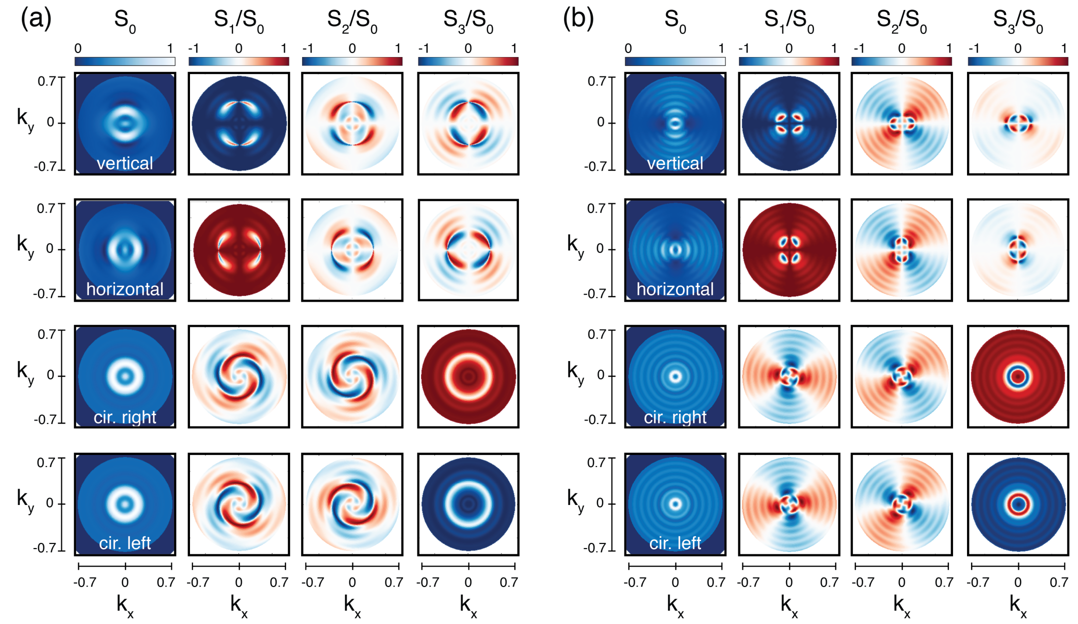

Fig. S 1. Calculated angle-resolved Stokes parameters for BE_{440} in (a) and BE_{600} in (b), under linearly (first two rows) and circularly polarized light (second two rows), corresponding to the measurements in Fig. 3. For both figures $|A| = 0.1\ \mu\mathrm{m}^{-1}$, $\varphi = \pi/2$, $l_c = 10p$, $a = 149$ nm for (b) and $a = 266$ nm for (d).

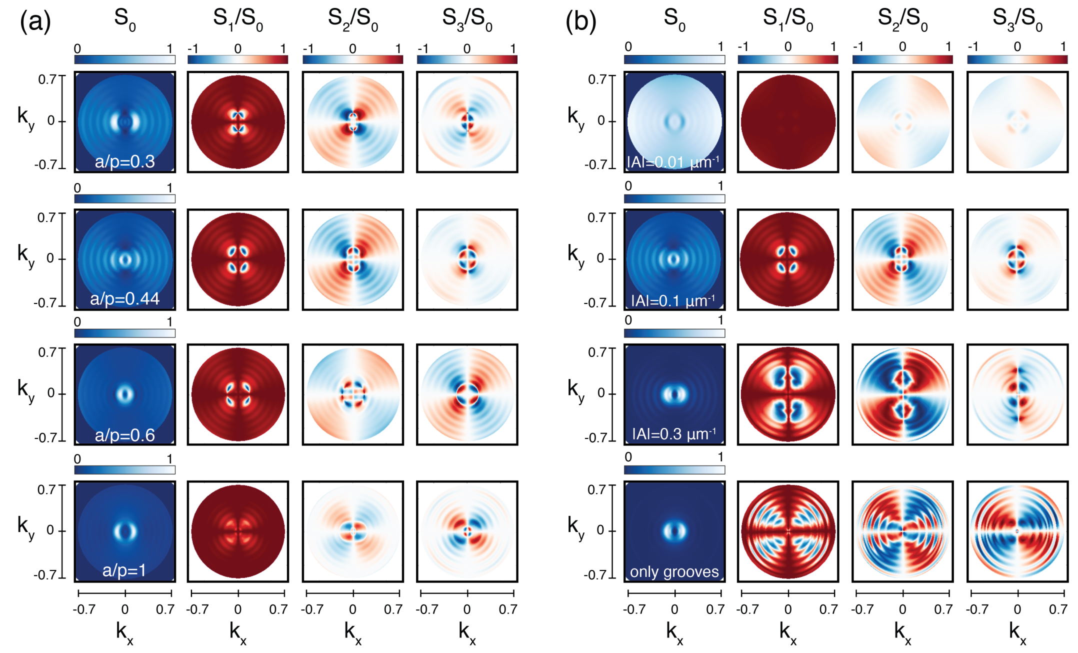

Fig. S 2. Calculated angle-resolved Stokes parameters for BE_{600} illuminated by horizontally polarized light. In (a) as a function of the ratio of a to pitch p with $|A| = 0.1\ \mu\mathrm{m}^{-1}$, and in (b) as a function of $|A|$ with $a/p = 0.44$. For all figures $\varphi = \pi/2$ and $l_c = 10p$.
